# Syngeneic Cardiac and Bone Marrow Stromal Cells Display Tissue-Specific microRNA Signatures and microRNA Subsets Restricted to Diverse Differentiation Processes

**DOI:** 10.1371/journal.pone.0107269

**Published:** 2014-09-18

**Authors:** Viviana Meraviglia, Valerio Azzimato, Luca Piacentini, Mattia Chiesa, Rupesh K. Kesharwani, Caterina Frati, Maurizio C. Capogrossi, Carlo Gaetano, Giulio Pompilio, Gualtiero I. Colombo, Alessandra Rossini

**Affiliations:** 1 Laboratory of Vascular Biology and Regenerative Medicine, Centro Cardiologico Monzino IRCCS, Milano, Italy; 2 Center for Biomedicine, European Academy Bozen/Bolzano (EURAC) (affiliated Institute of the University of Lübeck), Bolzano, Italy; 3 Department of Pharmacology, Chemotherapy and Medical Toxicology, Università degli Studi di Milano, Milano, Italy; 4 Laboratory of Immunology and Functional Genomics, Centro Cardiologico Monzino IRCCS, Milano, Italy; 5 Department of Electrical Computer and Biomedical Engineering, Università degli Studi di Pavia, Pavia, Italy; 6 CISTAC, Dipartimento di Medicina Interna e Scienze Biomediche, Università di Parma, Parma, Italy; 7 Laboratory of Vascular Pathology, Istituto Dermopatico dell’Immacolata IRCCS, Roma, Italy; 8 Division of Cardiovascular Epigenetics, Department of Cardiology, Goethe University, Frankfurt am Main, Germany; 9 Department of Clinical Sciences and Community Health, Università degli Studi di Milano, Milano, Italy; Tokai University, Japan

## Abstract

MicroRNAs are key modulators at molecular level in different biological processes, including determination of cell fate and differentiation. Herein, microRNA expression profiling experiments were performed on syngeneic cardiac (CStC) and bone marrow (BMStC) mesenchymal stromal cells cultured in standard growth medium and then *in vitro* exposed to adipogenic, osteogenic, cardiomyogenic and endothelial differentiation media. Analysis identified a tissue-specific microRNA signature composed of 16 microRNAs that univocally discriminated cell type of origin and that were completely unaffected by *in vitro* differentiation media: 4 microRNAs were over-expressed in cardiac stromal cells, and 12 were overexpressed or present only in bone marrow stromal cells. Further, results revealed microRNA subsets specifically modulated by each differentiation medium, irrespective of the cell type of origin, and a subset of 7 microRNAs that were down-regulated by all media with respect to growth medium. Finally, we identified 16 microRNAs that were differentially modulated by the media when comparing the two tissues of origin. The existence of a tissue-specific microRNA signature surviving to any differentiation stimuli, strongly support the role if microRNAs determining cell identity related to tissue origin. Moreover, we identified microRNA subsets modulated by different culture conditions in a tissue-specific manner, pointing out their importance during differentiation processes.

## Introduction

microRNAs (miRs) are 21–23 nucleotide non-coding RNA molecules, which modulate the stability and/or the translational efficiency of messenger RNAs (mRNA). Since miRs may target multiple transcripts and individual transcripts may be subject to multiple miR regulation, it is easy to appreciate that most biological processes are, at least in part, under the influence of miRs [1]. Interestingly, evidences have been provided that miRs can have binding motifs also located in the promoter regions [2] or into the sequence of long non-coding RNA [3], thus enormously extending their possible functions. Importantly, miR have been involved in pluripotency maintenance [4], cell proliferation and differentiation [5], epithelial to mesenchymal transition [6], senescence [7], and apoptosis [8].

Due to their wide role in cell process regulation, miR have gained popularity also as tools that are able to promote direct cell to cell phenotypic conversion as well as adult cell reprogramming into pluripotent stem cells. In fact, it has been recently demonstrated that miRs have the possibility to induce fibroblast differentiation into cardiomyocyte-like cells [9] and to facilitate, in concert with specific transcription factors, the conversion of adult human fibroblasts into neurons [10] or cardiomyocyte-like cells [11]. In addition, miRs might promote adult cell reprogramming into pluripotent cells [12], [13], although further work has to be done to understand whether miRs alone are sufficient to reprogram somatic cells into stem cells or other type of specialized cells. Nevertheless, being able to regulate and, possibly, to fine tune cell fate, miRs appear as a new frontier for application in regenerative medicine.

We recently characterized a population of cardiac mesenchymal stromal cells (CStC) from adult human atrial appendages [14]. This fibroblast-like, plastic-adherent cell population shared the expression of mesenchymal-associated antigens (*i.e.* CD105, CD73, CD29, and CD44) with stromal cells from other tissues. Nevertheless, it also exhibited specific properties, like a more pronounced ability than stromal cells of bone marrow origin (BMStC) of differentiating towards cardiomyocyte and endothelial phenotypes both in *in vitro* and *in vivo* settings. Being easily obtainable from small biopsy specimens and amplifiable *in vitro* up to therapeutically suitable numbers, CStC appear as a cell population useful for regenerative medicine applications.

In order to contribute in clarifying miR role in the definition of stromal cell identity and fate we: (1) isolated CStC and BMStC from syngeneic donors and cultured them in standard growth conditions (2) exposed them to four media previously used in literature to promote their differentiation into adipocyte, osteocyte-, endothelial-, cardiomyocyte-like cells and (3) analyzed their miR profile before and after differentiation treatments.

Specific aims of the present work were to: (1) identify a tissue-specific miR expression signature which was not influenced *in vitro* by differentiation media; (2) identify miR subsets specifically modulated by each differentiation medium, independently from the cell type of origin; and (3) identify those miRs that are differently modulated by the media between the two cell types.

To do so, we used a two-factor experimental approach that allowed us to ascertain miRs that unequivocally discriminated the cell type of origin, miRs that are similarly modulated by differentiation media in both cell types, and miRs that are differentially modulated by the media in the cell types. In addition, bioinformatics tools were used to relate miR expression to their predicted and/or validated mRNA targets in order to propose an interpretation of the results in terms of functional consequences on cell function, stemness and regenerative potential.

## Materials and Methods

### Ethics Statement

CStC and BMStC were obtained respectively from right auricle and sternal marrow samples of the same donor patients (*n* = 4) undergoing cardiac surgery, after approval by the Centro Cardiologico Monzino (Milano, Italy) Local Ethics Committee and signed informed consent. Experiments were conducted in accordance to the principles expressed in the Declaration of Helsinki. All data were analyzed anonymously.

### CStC and BMStC isolation and culture

CStC and BMStC were isolated as described in [14]. Briefly, CStC were enzymatically isolated from small auricle fragments using 3 mg/ml collagenase (Serva) and cultured in standard growth medium (GM), composed of Iscove’s Modified Dulbecco’s Medium (IMDM, Lonza) supplemented with 20% fetal bovine serum (FBS, Hyclone), 10 ng/ml basic Fibroblasts Growth Factor (bFGF, R&D), 10.000 U/ml Penicillin/Streptomycin (Invitrogen), 20 mM L-Glutamine (Sigma-Aldrich). BMStC obtained from 5 ml of heparinized bone marrow were separated by stratification on Ficoll gradient and cultured in the same GM.

### *In vitro* cell differentiation

CStC and BMStC were plated at a density of 5000 cells/cm^2^ and exposed to standardize differentiation-inducing media for 21 days. Media were changed twice a week. Adipogenic and osteogenic differentiation were achieved following standard *in vitro* protocols [15]. Endothelial differentiation was stimulated by culturing the cells in Endothelial Growing Medium 2 (EGM-2, Lonza), while differentiation towards the cardiomyogenic lineage was stimulated by culturing the cells in a medium composed by IMDM with 5% FBS (EuroClone), 5 µM All-Trans Retinoic Acid (ATRA) and 5 µM phenylbutyrate (PB, Sigma-Aldrich), as previously described [14]. After exposure to adipogenic, osteogenic and cardiomyogenic media, cells stopped proliferating, so they were not passaged anymore and the media were changed twice a week. On the contrary, cells exposed to endothelial and growth medium kept on proliferating and were consequently trypsinized and passaged when reaching 80–90% of confluence (usually every two or three days).

### Intracellular lipid staining by Oil-Red O

The accumulation of lipid droplets was evaluated by Oil-Red O staining [16]. The quantification of Oil-Red O positive cells was obtained by counting the number of positive cells *vs.* total number of cells.

### Von Kossa staining

The production of mineralized matrix was evaluated by von Kossa staining as previously described [16].

### Alkaline phosphatase assay

The presence of alkaline phosphatase was evaluated according to manufacturer’s instructions (AnaSpec).

### Ac-LDL-DiI uptake

Cells were incubated with 1,1-dioctadecyl-3,3,3,3-tetramethylindocarbocyanine-labeled acetylated LDL (Ac-LDL-DiI, Biomedical Technologies) as indicator of endothelial cells differentiation [16], [17]. After fixation with 4% PFA, cells were counterstained with Hoechst 33258 nuclear and observed with a Zeiss microscope equipped for epifluorescence.

### Flow cytometry

Cells were detached using 0.02% EDTA solution (Sigma-Aldrich) and stained with VEGFR2 PE-conjugated antibody (R&D) for 10 min at room temperature in the dark. In a different tube, cells were incubated with the correspondent IgG isotype, conjugated with the same fluorochrome used for the primary antibody. Cells were analyzed using a FACSCalibur (Becton–Dickinson) equipped with Cell-Quest Software v2.4.

### Immunofluorescence

α-sarcomeric actin expression was detected by incubation with specific primary antibody (AbCam) and FITC-conjugated secondary antibody (Jackson Immunoresearch). Nuclei were counterstained with Hoechst 33258 (Sigma-Aldrich) [14].

### microRNA profiling

Total RNA was extracted from cells using TRIzol reagent (Invitrogen) in accordance to the manufacturer’s instruction. Total RNA concentration and purity were evaluated by a NanoDrop 1000 spectrophotometer (Thermo Scientific), while RNA integrity was assessed with an Experion electrophoresis system and RNA High Sense Analysis Kit (Bio-Rad). Only high quality RNAs, with A260/A280 and A260/A230 ratios >1.8 and a RQI≥9.5/10, were used for subsequent investigations. Comparative miR expression profiling was carried out using the TaqMan Low Density Array Human MicroRNA Panel (Applied Biosystems), according to the manufacturer’s instructions, using a 7900TH Real Time PCR System (Applied Biosystems).

Prior to the analysis, probes were renamed and reannotated according to miRBase Release 20 (http://www.mirbase.org) [18]. This allowed us identifying 360 target sequences unique to human miRs, discarding probes for tRNA, snoRNA, and misannotated sequences. Expression analysis and quality control of TaqMan Arrays were performed using the ExpressionSuite Software v1.0.3 (Applied Biosystems). All Ct values reported as greater than 40 or as not detected were changed to 40 and considered a negative call. Raw expression intensities of target miRs were normalized for differences in the amount of total RNA added to each reaction using the mean expression value of all expressed miRs in a given sample [19]. Relative quantitation of miR expression was performed using the comparative Ct method (ΔΔCt). MiRs were deemed as non informative and filtered out when the percentile of negative calls exceeded 6 (20% of the samples): thus, the number of miRs considered for subsequent analysis was 306.

### Reverse Transcription – Real-time Polymerase Chain Reaction Analysis (RT-qPCR)

For gene expression analysis 1 µg of total RNA was reversely transcribed using the Superscript III reverse transcriptase (Invitrogen). cDNA was amplified by SYBR-GREEN quantitative PCR on an iQ5 Cycler (Bio-Rad). The following primers were used to detect α-sarcomeric actin transcript expression: forward 5′-TGTCCTGAGACACTCTTC-3′; reverse 5′-TGATGCTATTGTAAGTTGTT-3′. Samples were normalized to the Ct value of GAPDH, chosen as internal control. Relative quantitation was performed using the ΔΔCt method. Fold changes in gene expression were estimated as 2^(−ΔΔCt)^ [20].

To validate array-derived expression data, individual miR expression was analyzed using specific single-assay primers and target probes (Applied Biosystems) for miR-1, 126-5p, 133b, 135a-5p, 142-5p, 146a-5p, 155-5p, 184, 204-5p, and 222-3p. Reverse-transcription and real-time reactions were performed according to the manufacturer’s instructions, using a 7900TH Real Time PCR System (Applied Biosystems). Raw expression intensities of target miRs were normalized using the mean expression value of RNU44 and U6 RNA in any given sample. Relative quantitation was performed using the ΔΔCt method.

### Statistical analysis

The MeV v4.9.0 software [21] was used for the primary statistical and for unsupervised hierarchical clustering analyses. The GraphPad Prism v5.03 software was used for post-hoc analyses. Array data were analyzed by 2-factor ANOVA, calculating the *P-*values based on the F-distribution. In order to control for the false discovery rate (FDR), a *q-*value was estimated for each gene [22], both for the effects of the two factors and for the interaction *P*-value, using the QVALUE v1.36.0 implemented in Bioconductor v2.13 software package. The *q-*value was used as a FDR-based measure of significance and the threshold α was set to ≤0.01. Bonferroni post-hoc test was used to compare the effects of each medium within cells (*vs.* GM) and between cells (CStC *vs.* BMStC) and adjusted *P*-values <0.05 were considered statistically significant. Linear regression analysis and calculation of Pearson correlation coefficients were performed to relate array to singleplex qPCR expression data.

### Bioinformatics analysis

Queries for miR target prediction with three different algorithms (miRanda, PITA and TargetScan) was performed using the web-based tool MAGIA [23], applying stringent score filters (-12 for PITA, 500 for miRanda). Experimentally validated microRNA-target interactions were retrieved from the miRTarBase repository Release 4.5 [24]. The gene lists generated by these queries were exploited for gene-annotation (Gene Ontology, GO, terms and KEGG pathways) enrichment analysis using the web-based application DAVID 6.7 [25]: the EASE score (a conservative adjustment of the Fisher Exact *P*-value) threshold was set to 0.005 for GO terms and 0.05 for KEGG pathways. Redundant GO terms were removed using the web-based tool REViGO [26], with an allowed similarity threshold of 0.5. Irrelevant gene sets were removed manually.

Functional analysis of validated target genes for those miRs showing a statistically significant interaction between tissue and medium was performed using the Cytoscape [27] plugin ClueGO v2.1.2 [28], which allows analyzing and visualizing non-redundant biological terms for large lists of genes in a functionally grouped network. The network is created with kappa statistics, which reflects the relationships between the terms based on the similarity of their associated genes. ClueGO enrichment was calculated as a two-sided (enrichment/depletion) test based on the hypergeometric distribution, correcting for multiple testing by the Benjamini-Hochberg method. Percentage for cluster specificity was fixed to ≥55% and kappa score threshold for functional grouping to ≥0.3. Terms with an adjusted *P*-value<0.001 were selected for network visualization.

## Results

**Figure 1:**
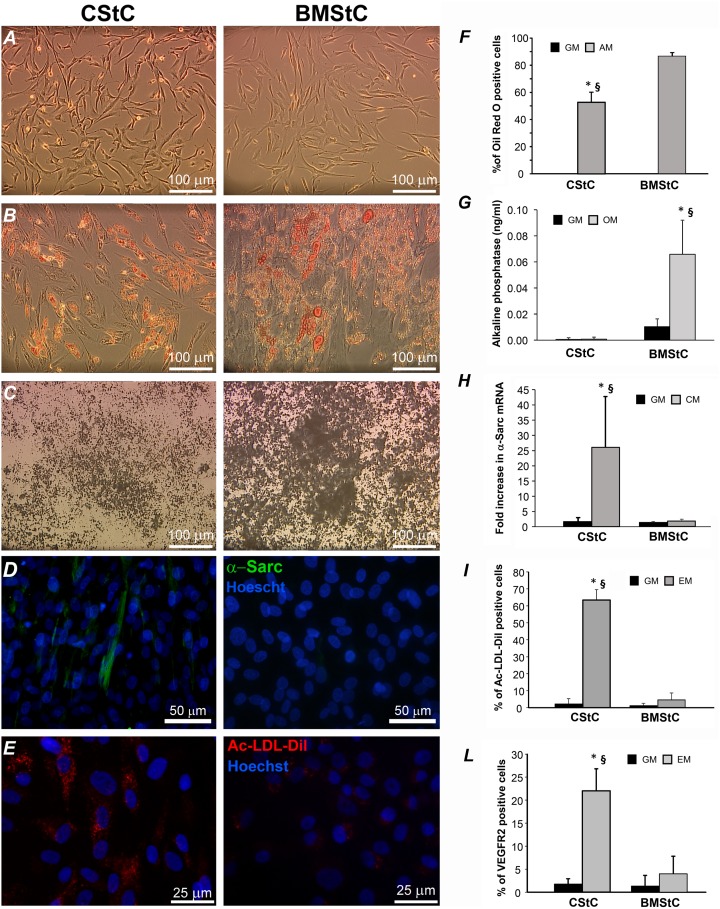
Morphology and response to *in vitro* differentiation. (***A***) Cardiac (CStC) and Bone Marrow (BMStC) Stromal Cells cultured in standard growth medium (GM). (***B***) CStC and BMStC exposed to adipogenic media (AM) show intracellular lipid accumulation as evidenced by Oil-red O staining. (***C***) Von Kossa staining of CStC and BMStC after osteogenic treatment: mineralized matrix is visualized by black dots. (***D***) Immunostaining for α-sarcomeric actin, a marker of cardiomyogenic differentiation. (***E***) Ac-LDL uptake assay: red in cytoplasm represents DiI-labeled acetylated LDL. Original magnifications: 10× for panels A, B, and C, and 40× for panels D and E. (***F***) Percentage of CStC and BMStC positive to Oil-red O staining in GM and after exposure to AM. (***G***) Accumulation of alkaline phosphatase was evaluated in CStC and BMStC cultured in GM and exposed for 21 days to osteogenic medium (OM). (***H***) RT-qPCR analysis for α-sarcomeric actin expression in CStC and BMStC after 3 weeks culture in GM and cardiomyogenic medium (CM). (***I***) Bar graph showing quantitative results for the Ac-LDL-DiI uptake in CStC and BMStC cultured in GM and exposed to endothelial medium (EM). (***L***) Representative flow cytofluorograms and bar graph indicating the percentage of VEGFR2 positive cells evaluated by FACS expression in CStC and BMStC before and after 3 weeks of EM culture. All the bar graphs show the mean values of 3 independent experiments ± SD (unpaired Student *t*-test: *P<0.001 *vs.* corresponding GM, §*P*<0.001 *vs.* BMStC).

Stromal cells obtained from different tissues show similar morphology and immunophenotype but different plastic properties [29]. Consistently with our previous results [14], CStC exhibited lower ability to gain both adipocyte and osteocyte features, esteemed by Oil-red O, Von Kossa staining and alkaline phosphatase measurements (Figure 1A, B, F, G). We also confirmed that CStC [30] displayed greater propensity to differentiate into cardiomyocyte-like and endothelial-like cells when compared to BMStC [31]. This was demonstrated after 21 days of differentiating treatment by (*i*) a higher expression of α-sarcomeric actin (evaluated by immunofluorescence and RT-qPCR, Figure 1D, H), (*ii*) a more efficient accumulation of Ac-LDL (Figure 1E, I), and (*iii*) a higher number of VEGFR2 positive cells (Figure 1L). Of note, both CStC and BMStC in growth medium (GM) exhibited negligible spontaneous differentiation. This different behavior is likely due to distinct molecular networks activated or repressed in the two cell populations despite their phenotypical similarity. To prove this hypothesis, miR expression profiles were evaluated by low-density microRNA TaqMan array in both CStC and BMStC exposed for 21 days to standard culture conditions (GM) or to four differentiation media, namely Adipogenic (AM), Osteogenic (OM), Cardiomyogenic (CM), and Endothelial (EM) Media. Differentially expressed genes were sought by performing a 2-way ANOVA, the two factors being the tissue of origin and the medium to which cells were exposed. Results obtained by this analysis led us to identify a grand-total of 115 significantly modulated miRs (after correction for multiple comparisons with a FDR≤0.01) either by the tissue of origin, or by the differentiation media, or by the interaction between the two factors (Table S1).

**Figure 2:**
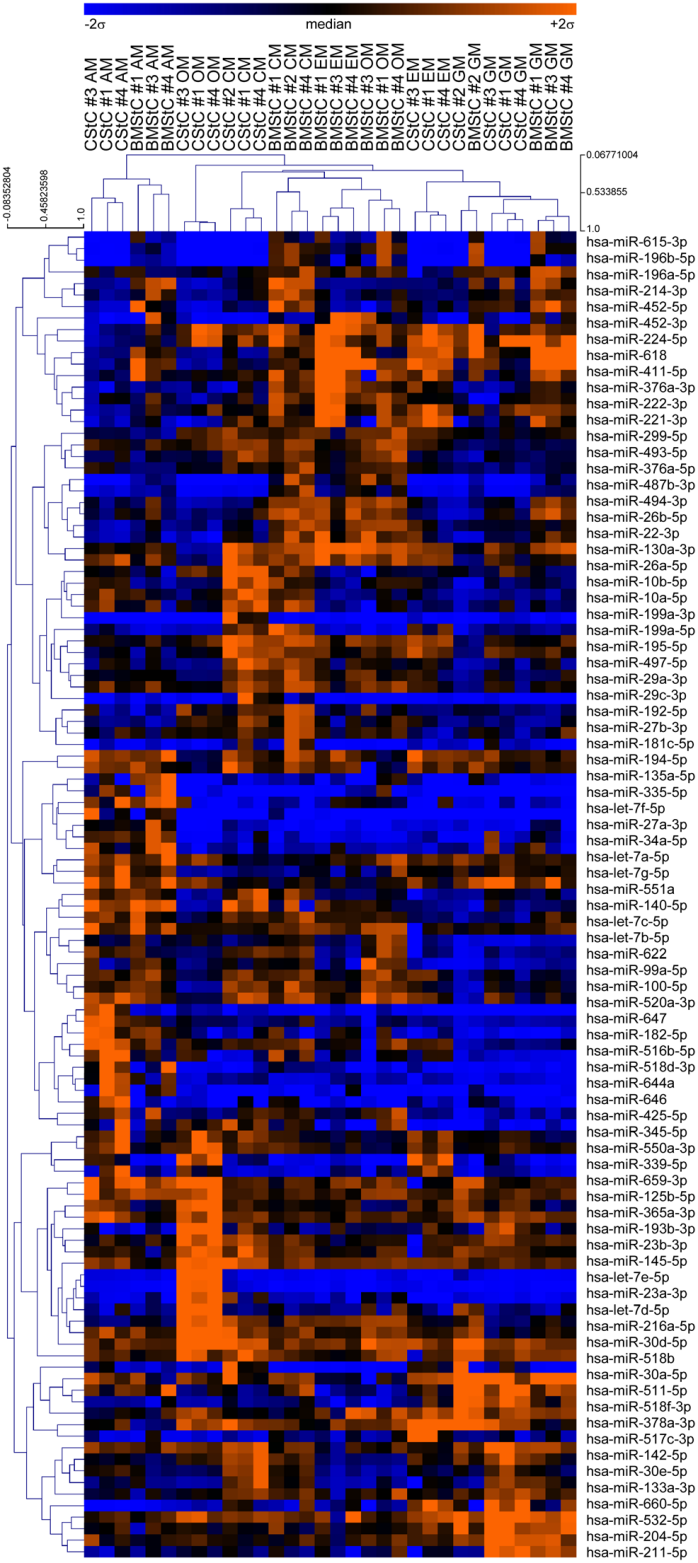
Unsupervised hierarchical clustering of miRs influenced by tissue of origin and/or differentiation media and/or interaction. Two-way ANOVA identified 115 miRs significantly modulated (FDR≤0.01). Samples and miRs were clustered using Pearson’s correlation (centered) and average linkage method. Each combination of cell type and differentiation medium was grouped in distinct clusters. The relative expression level of each miR is represented with a blue, black, and orange color scale, ranging from samples with −2 to +2 standard deviations from the mean (blue indicates below median; black, equal to, and orange, above median). CStC, Cardiac and BMStC, Bone Marrow Stromal Cells; GM, growth medium; AM, Adipogenic Medium; OM, Osteogenic Medium; CM, Cardiomyogenic Medium; EM, Endothelial Medium.

An unsupervised hierarchical clustering (Figure 2) revealed that the expression profile of these 115 miRs was able to fully discriminate medium-differentiated cells, *i.e.* cells of the same origin cultured in the same conditions were grouped in distinct nodes of the sample dendrogram. Interestingly, also CStC and BMStC cultured in GM belonged to the same main cluster, but a deeper insight revealed that only cells #1, 3, and 4 clustered according to the tissue of origin: CStC and BMStC from patient #2 grouped together in a distinct sub-cluster. This might be due to specific characteristics or different genetic background of patient #2, although the survey of his clinical records did not evidence anything that could explain his different behavior.

### Tissue-specific miR profiles

**Figure 3:**
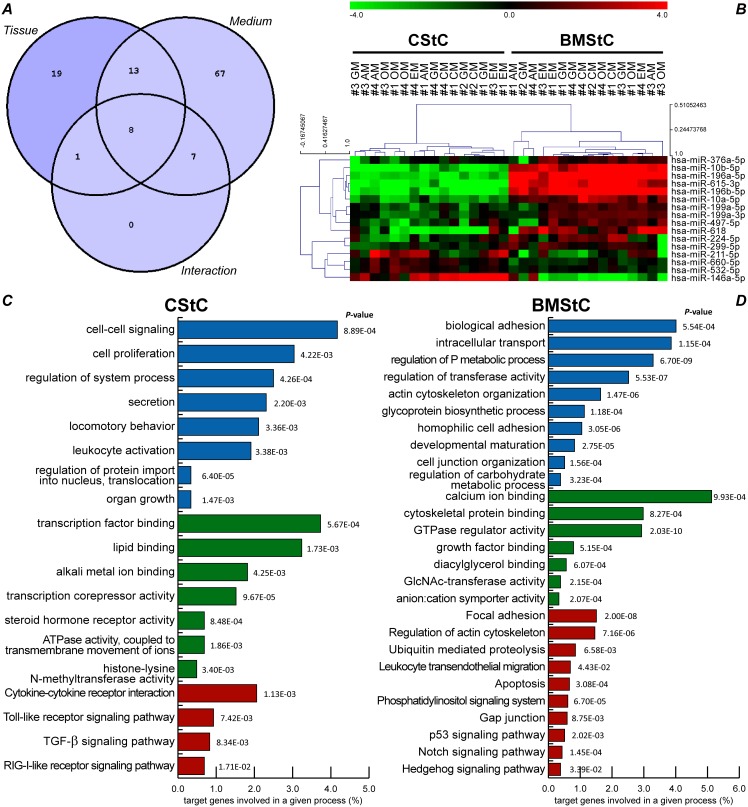
miR tissue signature and its potential functional implications. (***A***) A Venn diagram of the 115 significantly modulated miRs helps indentifying those exclusively affected by the tissue of origin (upper left segment) or by one or more differentiation media (upper right), and those influenced by both factors (upper mid intersection) or by the interaction between the two (lower intersection segments). (***B***) Unsupervised hierarchical clustering defining the miR tissue signature composed by 16 miRs, distinctive of the tissue of origin and with at least a 2-fold difference. Log_2_ transformed miR expression signals were centered by median values and samples and miRs were clustered using Pearson’s correlation (centered) and average linkage method, with leaf order optimization. The dendrogram above shows that this signature is able to divide Cardiac (CStC) and Bone Marrow (BMStC) Stromal Cells in two distinct clusters, irrespective of the culture media exposure. The relative expression level of each miR is represented with a green, black, and red color scale (green indicates below, black equal to, and red above median). (***C***) Gene-annotation enrichment analysis revealed GO biological processes (blue bars), molecular functions (green), and KEGG pathways (red) potentially and exclusively targeted by CStC tissue-specific miRs. The x-axis represents the percentage of genes belonging to a given GO or KEGG term with respect to the total predicted and validated targets. EASE score *P*-values are reported for every term. (***D***) Gene categories potentially targeted by BMStC tissue-specific miRs, as revealed by gene-annotation enrichment analysis. GM, growth medium; AM, Adipogenic Medium; OM, Osteogenic Medium; CM, Cardiomyogenic Medium; EM, Endothelial Medium.

Among the 115 differentially modulated miRs, 41 miRs were dependent on the tissue of origin. Accordingly, the unsupervised hierarchical clustering showed that these 41 miRs were able to divide CStC and BMStC into two different clusters independently of the culture conditions (Table S1). We then used a Venn diagram (Figure 3A) to visualize which miRs were exclusively influenced by the tissue of origin, excluding those miRs that were modulated also or solely by the culture medium and/or by the interaction between the two factors. The remaining subset was composed of 19 miRs that were independent from and unmodulated by any differentiation stimuli. To further refine this tissue-specific miR profile, we excluded 3 miRs that showed a mean fold difference between CStC and BMStC≤|±2| (*i.e.* miR-214-3p, 324-3p, and 365a-3p). This allowed us to identify two tissue specific miR signatures (Figure 3B), which included: 4 miRs that were significantly overexpressed in CStC (miR-146a-5p, 211-5p, 532-5p, and 660-5p); 8 miRs overexpressed in BMStC (miR-10a-5p, 199a-3p, 199a-5p, 224-5p, 299-5p, 376a-5p, 497-5p, and 618) plus 4 BMStC-specific miRs that were virtually absent in CStC (miR-10b-5p, 196a-5p, 196b-5p, and 615-3p).

Importantly, gene-annotation enrichment analysis, conducted on both predicted and validated targets of the two miR signatures, showed that several pathways and gene categories are targeted by both CStC and BMStC specific miRs, *e.g.* calcium, insulin, MAPK, ErbB, Jak-STAT, mTOR, and Wnt signaling pathways (not shown). Conversely, this analysis (Figure 3C and D) revealed a number of distinct unique GO biological processes (blue-colored bars), molecular functions (green), and KEGG pathways (red), which are potentially targeted by the signature miRs in either cell populations.

### Medium-specific miR profiles

**Figure 4:**
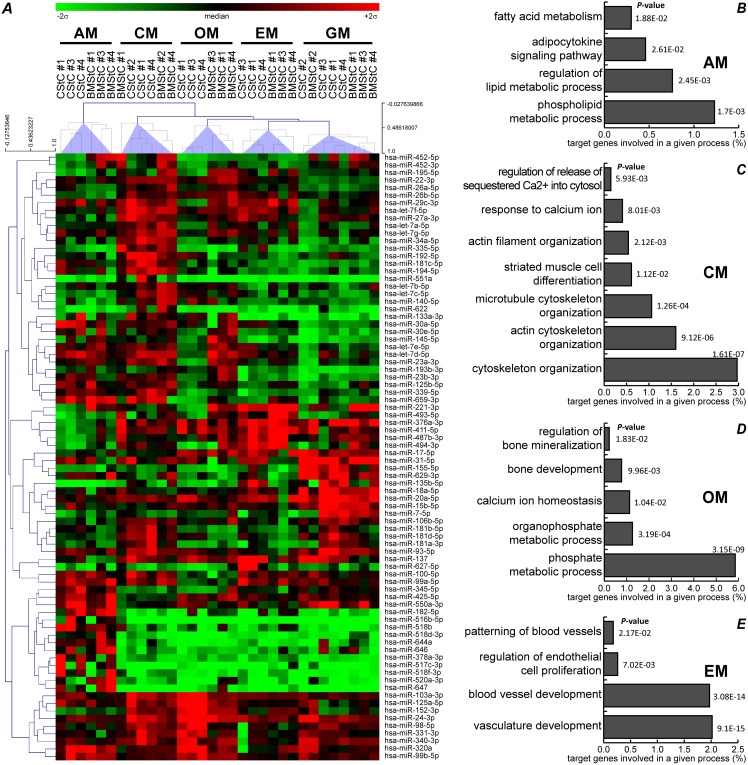
miRs specifically influenced by differentiation media. (***A***) Heatmap representing the expression of 80 miRs significantly modulated by differentiation stimuli (FDR≤0.01) independently from the tissue of origin. Unsupervised hierarchical analysis groups in five distinct clusters both Cardiac (CStC) and Bone Marrow (BMStC) Stromal Cells exposed to the same medium, as highlighted by translucent purple wedges drawn from the five main nodes. Clustering was done using Pearson’s correlation (centered) and average linkage method. The relative expression level of each miR is represented with a green, black, and red color scale (as in Figure 3). Gene-annotation enrichment analysis showed relevant GO biological processes and KEGG pathways potentially targeted by miRs modulated by (***B***) Adipogenic Medium (AM), (***C***) Osteogenic Medium (OM), (***D***) Cardiomyogenic Medium (CM), and (***E***) Endothelial Medium (EM). EASE score *P*-values are reported for every term.

Two-way ANOVA identified 95 miRs that were significantly modulated by the differentiation media (Table S1). The Venn diagram analysis (Figure 3A) showed that 13 miRs were influenced by both the tissue of origin and the media (upper intersection area), *i.e.* that they were differentially expressed between BMStC and CStC and that one or more media modulated their expression similarly in both cell types. In addition, the Venn diagram revealed 67 miRs that were exclusively influenced by differentiation media, independently from the tissue of origin (*i.e.* no significant differential expression between the cell types, but a significant modulation in the same direction and of a similar extent in both CStC and BMStC). Importantly, unsupervised hierarchical clustering indicated that these 80 miRs were able to fully discriminate cells exposed to the same medium, independently from their tissue of origin (Figure 4A).

Post-hoc tests, conducted comparing each differentiation medium with the GM, combining BMStC and CStC data, allowed identifying smaller subsets of miRs specifically modulated in each condition (Table 1). Among them, miR-7-5p, 15b-5p, 18a-5p, 20a-5p, 31-5p, 155-5p, and 629-3p were significantly down-regulated by all media in both cells, while other subsets were specifically modulated by one or more differentiation conditions, with a fold change (FC) ≥|±2| in most cases (Table 1).

**Table 1:** MiR subsets specifically modulated by differentiation media *vs.* growth medium in both CStC and BMStC.

microRNA	FC *vs.* GM				*P*-values†			
	AM	OM	CM	EM	AM	OM	CM	EM
**miR-629-3p**	−1.7	−5.5	−2.6	−3.9	*	***	***	***
**miR-15b-5p**	−2.2	−1.8	−2.4	−2.3	**	*	***	***
**miR-20a-5p**	−2.4	−1.8	−1.8	−1.6	***	**	**	*
**miR-18a-5p**	−2.7	−2.7	−2.5	−2.1	***	***	***	**
**miR-31-5p**	−2.9	−3.7	−2.3	−2.8	***	***	***	***
**miR-7-5p**	−4.4	−2.6	−3.3	−4.4	***	**	***	***
**miR-155-5p**	−6.6	−11.3	−4.5	−4.2	***	***	***	***
**miR-133a-3p**		12.1	13.2		*ns*	*	**	*ns*
**miR-659-3p**	5.4		3.4		***	*ns*	**	*ns*
**miR-222-3p**		2.8	3.1		*ns*	*	**	*ns*
**miR-29c-3p**			2.2	2.2	*ns*	*ns*	*	*
**miR-17-5p**	−2.6		−2.7		***	*ns*	***	*ns*
**miR-135b-5p**	−35.3	−5.8			***	**	*ns*	*ns*
**miR-516b-5p**	11243				*	*ns*	*ns*	*ns*
**miR-647**	1995				***	*ns*	*ns*	*ns*
**miR-518f-3p**	42.1				***	*ns*	*ns*	*ns*
**miR-518b**	29.6				***	*ns*	*ns*	*ns*
**miR-517c-3p**	15.3				**	*ns*	*ns*	*ns*
**miR-182-5p**	13.7				***	*ns*	*ns*	*ns*
**miR-378a-3p**	13.4				***	*ns*	*ns*	*ns*
**miR-520a-3p**	7.8				**	*ns*	*ns*	*ns*
**miR-30a-5p**	4.3				***	*ns*	*ns*	*ns*
**miR-644a**	3.5				*	*ns*	*ns*	*ns*
**miR-137**	−2.5				*	*ns*	*ns*	*ns*
**miR-376a-3p**	−5.0				***	*ns*	*ns*	*ns*
**miR-193b-3p**		8.6			*ns*	*	*ns*	*ns*
**miR-145-5p**		6.6			*ns*	**	*ns*	*ns*
**miR-152-3p**		2.9			*ns*	***	*ns*	*ns*
**miR-125a-5p**		2.3			*ns*	***	*ns*	*ns*
**miR-320a**		1.8			*ns*	*	*ns*	*ns*
**miR-335-5p**			5.4		*ns*	*ns*	***	*ns*
**miR-181c-5p**			5.0		*ns*	*ns*	***	*ns*
**miR-23b-3p**			4.5		*ns*	*ns*	*	*ns*
**miR-34a-5p**			4.1		*ns*	*ns*	***	*ns*
**let-7c-5p**			3.7		*ns*	*ns*	**	*ns*
**miR-194-5p**			3.6		*ns*	*ns*	***	*ns*
**miR-140-5p**			2.9		*ns*	*ns*	*	*ns*
**miR-339-5p**			2.8		*ns*	*ns*	*	*ns*
**miR-23a-3p**			2.7		*ns*	*ns*	*	*ns*
**let-7a-5p**			2.7		*ns*	*ns*	*	*ns*
**miR-192-5p**			2.6		*ns*	*ns*	**	*ns*
**let-7f-5p**			2.5		*ns*	*ns*	***	*ns*
**let-7 g-5p**			2.4		*ns*	*ns*	*	*ns*
**let-7d-5p**			2.4		*ns*	*ns*	*	*ns*
**miR-550a-3p**			−2.2		*ns*	*ns*	*	*ns*
**miR-627-5p**				4.0	*ns*	*ns*	*ns*	***
**miR-493-5p**				3.6	*ns*	*ns*	*ns*	***
**miR-494-3p**				2.6	*ns*	*ns*	*ns*	***
**miR-411-5p**				1.9	*ns*	*ns*	*ns*	***
**miR-221-3p**				1.9	*ns*	*ns*	*ns*	***
**miR-93-5p**				−2.3	*ns*	*ns*	*ns*	*

† Bonferroni corrected *P*-values.

^*^ *P*<0.05;

^**^ *P*<0.01;

^***^ *P*<0.001. FC: fold-change.

CStC, Cardiac and BMStC, Bone Marrow Stromal Cells; AM, Adipogenic Medium; OM, Osteogenic Medium; CM, Cardiomyogenic Medium; EM, Endothelial Medium.

In agreement with the expected effect on cell phenotype, a look-up of gene-annotation enrichment analysis revealed that miRs exclusively modulated (*i*) by AM may target pathways related to the lipid metabolism, (*ii*) by CM processes linked to cytoskeleton organization and calcium handling, (*iii*) by OM phosphate homeostasis and mineralization, and (*iv*) by EM vessel and endothelial cell proliferation (Figure 4B).

### Interaction effects

**Figure 5:**
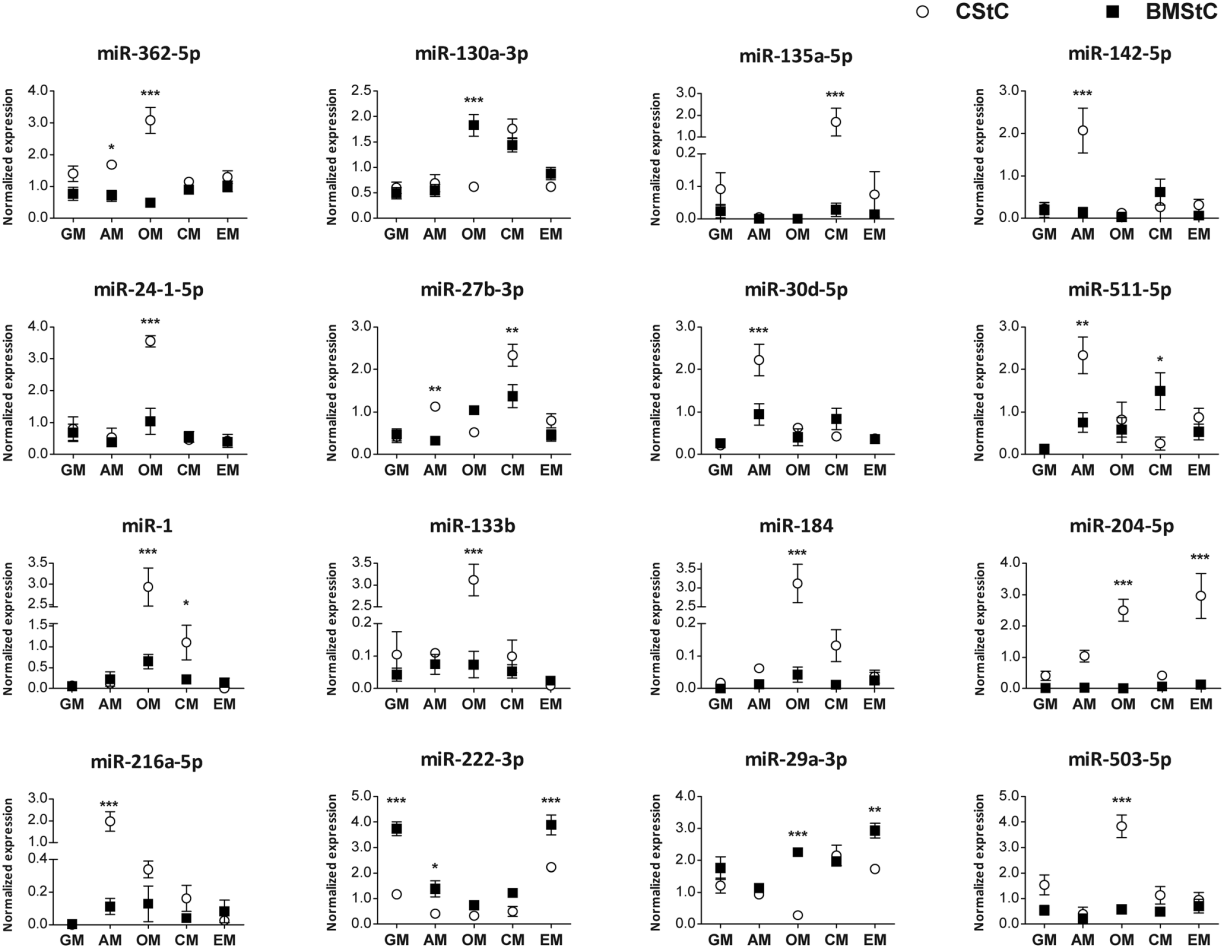
miRs for which the effect of the media differed between CStC and BMStC. Mean expression levels ± SEM are plotted for 16 miRs showing a significant interaction effect between tissue origin and media, at 2-way ANOVA, for both Cardiac (CStC) and Bone Marrow (BMStC) Stromal Cells (open circle and filled squares, respectively). Post-hoc comparison between CStC and BMStC indentified miRs differentially modulated by differentiation media. *P<0.05, **P<0.01, ***P<0.001. GM, growth medium; AM, Adipogenic Medium; OM, Osteogenic Medium; CM, Cardiomyogenic Medium; EM, Endothelial Medium.

Two-way ANOVA identified a group of 16 miRs, which showed a statistically significant interaction effect of the media on the cell type (Figure 5), *i.e.* miRs that were differentially modulated by one or more media in CStC *vs.* BMStC. In detail, the Venn diagram (Figure 3A) shows that the expression of one miR (362-5p) is influenced by both cell type and interaction, 7 by media and interaction (130a-3p, 135a-5p, 142-5p, 24-1-5p, 27b-3p, 30d-5p, 511-5p), and 8 by both factors (tissue and medium) and interaction (1, 133b, 184, 204-5p, 216a-5p, 222-3p, 29a-3p, 503-5p). Post-hoc analysis comparing CStC and BMStC allowed identifying subsets of miRs affecting differentiation in a cell-specific manner (Figure 5). Four subsets were distinctively modulated in CStC. The first subset comprises 5 miRs (142-5p, 216a-5p, 27b-3p, 30d-5p, and 511-5p) that were all up-regulated by AM in CStC to a significantly higher level than in BMStC (mean fold difference of 15.2, 17.5, 3.5, 2.3, and 3.1, respectively). A second subset consists of 7 miRs (1, 133b, 184, 204-5p, 24-1-5p, 362-5p, and 503-5p) up-regulated by OM in CStC and significantly higher than in BMStC (fold difference of 4.5, 42.4, 71.8, 432.7, 3.4, 6.3, and 6.6, respectively). The third subset includes 3 miRs (1, 135a-5p, and 27b-3p) up-regulated by CM in CStC to a significantly higher extent than in BMStC (fold difference of 5.0, 60.0, and 1.7, respectively). Finally, miR-204-5p was up-regulated in CStC also by EM, with a significant fold difference of 22.3 compared to in BMStC. Conversely, two miRs (130a-3p and 511-5p) were up-regulated by OM and CM, respectively, in BMStC but not in CStC (with significant fold differences of 2.9 and 5.7). In addition, miR-222-3p was down-regulated by all differentiation media but EM in both cell types, showing a significant higher expression in BMStC *vs.* CStC cultured in GM (3.2), AM (3.4), and EM (1.7); miR-29a-3p was down-regulated by OM in CStC (-4.3), and was significantly higher in BMStC than CStC cultured in OM (8.0) and EM (1.7).

**Figure 6:**
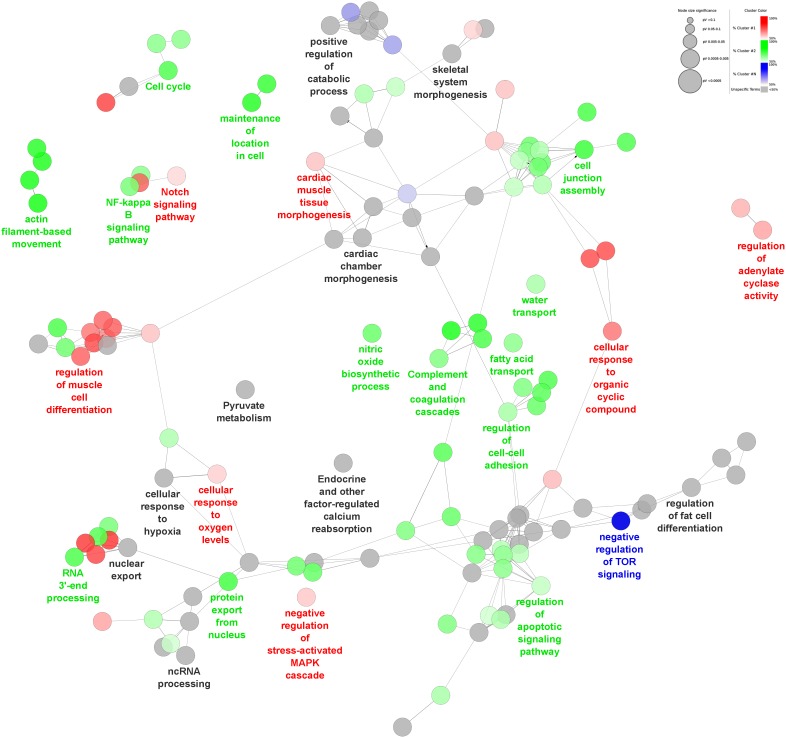
Differentiation cluster network based on functionally enriched GO terms and KEGG pathways. Functional differences of the over-represented GO Biological Processes (BP) and KEGG pathways for the adipogenic, osteogenic, and cardiomyogenic differentiation stimuli in CStC are shown. GO BP and pathway terms are represented as nodes and functional groups are linked to their biological function. Node labels show the most significant or relevant group gene set. Node size represents the term enrichment significance. Node color represents specific cluster membership, *i.e.* adipogenic (blue), osteogenic (green) or cardiomyogenic (red) differentiation clusters; grey nodes represent unspecific cluster related terms. Node color gradient (from lighter to darker) refers to the proportion of genes (ascending) of a specific differentiation cluster.

Experimentally validated targets of the four CStC-related miR subsets were analyzed for gene set enrichment and compared to construct a functionally grouped GO and KEGG pathway annotation network, which visualizes the differences of the four gene clusters (Figure 6). This analysis uncovered, besides functional terms not specific to any differentiation media, GO/KEGG gene sets explicitly targeted in CStC by miR subsets modulated by CM (cardiac muscle differentiation, response to cyclic compound second messenger system, MAPK signaling cascade), OM (fatty acid transport, cell cycle and apoptosis, cell adhesion), and AM (negative regulation of TOR signaling). No EM-specific gene set was evident. Table 2 summarizes the results of gene set enrichment analysis of the validated targets of the other miRs: miR-204-5p, consistently with its up-regulation by EM and OM in CStC, has an impact both on vasculature and skeletal system development. Target genes of miR-130a-3p are involved in the BMP and TGF-β signaling pathways and the regulation of osteoblast differentiation; those of mir-222-3p in cell cycle, response to stress and cytoskeleton organization; and those of miR-29a-3p in regulation of cell adhesion and regulation of ossification.

**Table 2:** Gene set enrichment analysis of 4 miRs differentially modulated by differentiation media in CStC *vs.* BMStC.

Term ID	Description	N*	%†	*P*-value‡
**miR-204**-**5p**				
GO:0001944	vasculature development	9	12.5	3.18E-05
GO:0051173	positive regulation of nitrogen compound metabolic process	13	18.1	6.61E-05
GO:0006357	regulation of transcription from RNA polymerase II promoter	13	18.1	2.08E-04
GO:0009611	response to wounding	11	15.3	2.60E-04
GO:0010558	negative regulation of macromolecule biosynthetic process	11	15.3	3.34E-04
GO:0001501	skeletal system development	8	11.1	9.82E-04
GO:0016044	membrane organization	8	11.1	2.71E-03
GO:0014070	response to organic cyclic substance	5	6.9	3.02E-03
GO:0018107	peptidyl-threonine phosphorylation	3	4.2	3.51E-03
GO:0048870	cell motility	7	9.7	4.07E-03
GO:0042127	regulation of cell proliferation	11	15.3	5.00E-03
hsa04210	Apoptosis	5	6.9	3.10E-03
hsa04621	NOD-like receptor signaling pathway	4	5.6	9.26E-03
hsa04060	Cytokine-cytokine receptor interaction	7	9.7	9.33E-03
hsa04520	Adherens junction	4	5.6	1.67E-02
**miR-130a-3p**				
GO:0007389	pattern specification process	8	32.0	2.02E-07
GO:0010604	positive regulation of macromolecule metabolic process	11	44.0	5.30E-07
GO:0045941	positive regulation of transcription	8	32.0	2.89E-05
GO:0030509	BMP signaling pathway	4	16.0	5.43E-05
GO:0042127	regulation of cell proliferation	8	32.0	2.37E-04
GO:0019220	regulation of phosphate metabolic process	6	24.0	1.14E-03
GO:0042592	homeostatic process	7	28.0	1.28E-03
GO:0045667	regulation of osteoblast differentiation	3	12.0	2.39E-03
GO:0006897	endocytosis	4	16.0	5.91E-03
hsa04350	TGF-beta signaling pathway	4	16.0	9.52E-04
hsa04144	Endocytosis	4	16.0	8.05E-03
hsa04060	Cytokine-cytokine receptor interaction	4	16.0	2.10E-02
**miR-222**-**3p**				
GO:0007049	cell cycle	30	10.9	7.41E-05
GO:0046907	intracellular transport	26	9.5	1.82E-04
GO:0006461	protein complex assembly	22	8.0	1.85E-04
GO:0010608	posttranscriptional regulation of gene expression	13	4.7	3.09E-04
GO:0010605	negative regulation of macromolecule metabolic process	26	9.5	9.34E-04
GO:0006396	RNA processing	21	7.7	1.32E-03
GO:0007017	microtubule-based process	13	4.7	1.53E-03
GO:0007010	cytoskeleton organization	18	6.6	1.54E-03
GO:0051173	positive regulation of nitrogen compound metabolic process	23	8.4	1.86E-03
GO:0006261	DNA-dependent DNA replication	6	2.2	3.23E-03
GO:0006357	regulation of transcription from RNA polymerase II promoter	24	8.8	3.80E-03
GO:0042493	response to drug	11	4.0	4.42E-03
GO:0033554	cellular response to stress	20	7.3	4.47E-03
hsa03030	DNA replication	5	1.8	4.42E-03
hsa04530	Tight junction	7	2.6	4.02E-02
**miR-29a-3p**				
GO:0001952	regulation of cell-matrix adhesion	4	5.7	2.25E-04
GO:0001775	cell activation	8	11.4	2.65E-04
GO:0051130	positive regulation of cellular component organization	6	8.6	1.22E-03
GO:0010035	response to inorganic substance	6	8.6	2.11E-03
GO:0010608	posttranscriptional regulation of gene expression	6	8.6	2.40E-03
GO:0006915	apoptosis	9	12.9	4.87E-03
GO:0030278	regulation of ossification	4	5.7	4.98E-03
hsa04510	Focal adhesion	8	11.4	4.27E-04
hsa04722	Neurotrophin signaling pathway	6	8.6	1.64E-03
hsa04512	ECM-receptor interaction	4	5.7	2.10E-02

*Number of genes belonging to a given GO or KEGG gene set with respect to the total validated targets for a given miR.

† Percentage of genes belonging to a given GO or KEGG gene set with respect to the total validated targets for a given miR.

‡ EASE score *P*-values.

### Singleplex RT-qPCR validation

**Figure 7:**
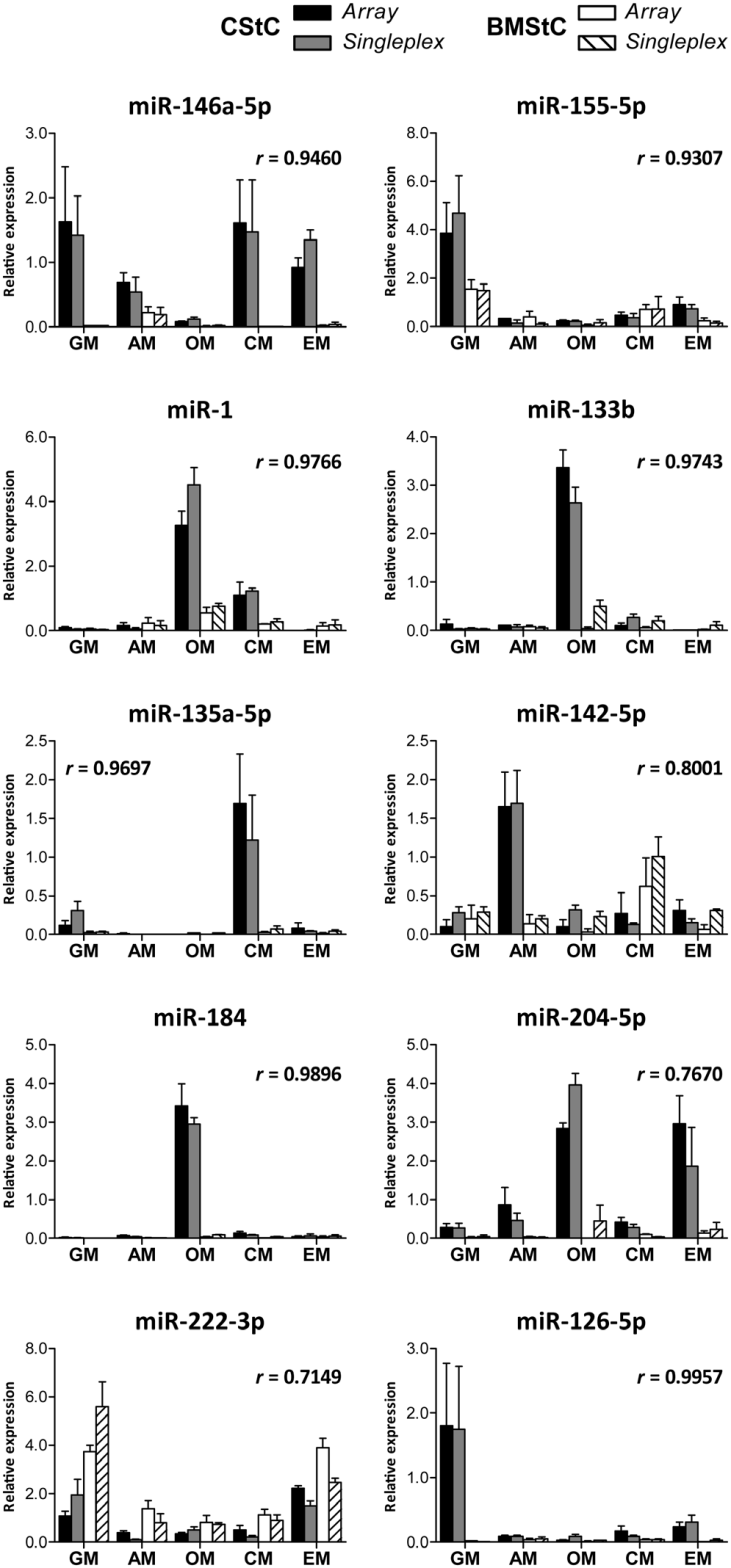
Validation by single assay RT-qPCR. Ten miRs (were validated by single-assay real-time PCR. The mean centered relative expression values are plotted respectively on y-axis, for the singleplex assays, and on the x-axis, for the arrays. All correlations were significant with *P*<0.001.

Single RT-qPCR experiments were performed for 10 miRs to validate data derived from the arrays (Figure 7). To cover all possible combinations, we chose one miR from the tissue signature (miR-146a-5p), one modulated by all media but not differentially expressed between the cell types (155-5p), seven with a significant interaction *q-*value and influenced by both tissue and medium (1, 133b, 135a-5p, 142-5p, 184, 204-5p, and 222-3p), and one miR that was not significantly different between cell types and among differentiation media (126-5p). Results fully confirmed the array data, as the Pearson coefficient was ≥0.70 and *P*-values<0.001 for every correlation analysis.

## Discussion

MicroRNAs have been suggested to be part of the molecular network responsible for cell identity regulation [14], [32]. In the present work, we verify that stromal cells obtained from different tissues retain unaltered the expression of miR subsets even after differentiation [14] and highlight significant influences of diverse differentiation strategies on miR expression profiles. Most interestingly, our results pointed out an interaction between culture media and tissue of origin in determining miR expression of a given cell type and affecting differentiation in a cell-specific manner.

MiRs included in the tissue signature remained unmodified after *in vitro* standard differentiation treatments. This observation has important consequences, confirming that miR-regulated pathways are involved in cell identity and fate determination. Among the 16 miRs constituting the BMStC specific signature, miRs-10a, 10b, 196a, 196b, 199a and 615 have a role in controlling cell cycle, proliferation and development [33], [34]. Of note, miR-146a, is highly expressed in proliferating cardiovascular precursors [35], consistently with its higher expression in CStC compared to BMStC. Importantly, GO analysis revealed that miR specifically overexpressed in BMStC might target processes involved in phosphorous metabolism, which is in line with the increased osteogenic ability of BMStC compared to CStC. On the other hand, miR specifically up-regulated in CStC can actually target transmembrane ion movement and toll-like receptor signaling pathways. These findings are in agreement with the ability of CStC to acquire some excitable cell properties [30] and to respond to HMGB-1 stimulation [36], respectively.

MiRs modulated only by differentiation stimuli independently from tissue origin were expressed at similar levels in CStC and BMStC cultured in GM and significantly modulated at a comparable extent and direction in the two cell populations after differentiation treatment. Within this group, a smaller subset of 7 miRs was significantly down-regulated by all the differentiating conditions. The down-modulation of miR-155, 20a-5p, and 18a-5p is consistent with the reduced proliferation ability observed during differentiation processes, in agreement with their involvement in cell proliferation and apoptosis suppression [37], [38]. Further, suppression of miR-7-5p and miR-31-5p is related to differentiation processes, such as osteogenesis [39], myogenic differentiation [40], and vascular development [41]. Other miRs appeared to be regulated mainly by one differentiation stimulus. Members of the let-7 family, together with miR-23a and 23b, were significantly up-regulated after cardiomyogenic treatment, in agreement with their reported role in cardiac differentiation [42] and cardiovascular processes [43]. Similarly, miR-320, 193 and 125a, which were significantly up-regulated after osteogenic treatment, have been associated with osteogenesis of mesenchymal cells in vitro [44], [45]. Conversely, the exposure of both cell types to EM resulted in a down-regulation of miR-93-5p, which has been related to angiogenesis [46]. Further, in line with their known role in adipogenesis, miR-30a-5p and 378a-5p were up-regulated by AM in both cell types: it has been demonstrated that during adipogenic differentiation miR-30a overexpression induces the activation of the key transcription factor PPARγ [47], which in turn positively regulates miR-378a-5p expression [48].

miRs that showed an interaction between the tissue origin and the differentiation media are those that differentially responded to the differentiation stimuli comparing CStC and BMStC. The four culture media up-regulated four partially overlapping miR subsets in CStC but not in BMStC. Gene set enrichment analysis showed that the three miRs (1, 27b-3p, and 135a-5p) associated with CM treatment of CStC has a profound impact on pathways related to the regulation of muscle differentiation. Of note, miR-133a was also significantly up-regulated by CM in both cells (see Table 1). This is in agreement with the known role played by miR-1 and miR-133, which are highly expressed in cardiac muscle cells and are critical regulators of muscle differentiation and proliferation [49], [50]. Consistently, miR-27b, which was also up-regulated by AM in CStC, has been shown to have an enhanced myocardial expression during heart development [51], and to impair human adipocyte differentiation by targeting PPARγ [52]. On the other hand, miR-135a-5p suppressed 3T3-L1 preadipocyte differentiation and adipogenesis through the activation of canonical Wnt/β-catenin signaling [53]. Overexpression of miR-142, another miR that we found upregulated by AM in CStC, has been shown to be inversely related to MAPK activity in cultured cardiac myocytes, inhibiting both survival and growth pathways and repressing multiple components of the NF-κB pathway, and to induce extensive apoptosis and cardiac dysfunction in a model of cardiac hypertrophy [54]. Conversely, miR-30d modulation by AM in CStC is consistent with its reported role as a key regulator of adipocyte development, by targeting the transcription factor RUNX2 and stimulating adipogenesis via the modulation of this major regulator of osteogenesis [47]. Interestingly, the expression of myomiRs like miR-1 and miR-133b [55] was increased in CStC compared to BMStC after osteogenic treatment, which is not surprising considering that this medium contained Dexamethasone, a drug known to partially induce cardiomyogenic differentiation of adult cardiac stem cells [56]. Among other miRs specifically up-regulated by OM in CStC, miR-184 has been shown to suppress proliferation and survival in tumor cells [57]. Importantly, the network analysis on functionally enriched pathways showed that gene clusters related to skeletal system morphogenesis or regulation of fat cell differentiation are not specifically modulated by any CStC-related miR subsets, whereas the only pathway specifically targeted by AM-related miRs was mTOR signaling, which in turn is known to promote adipogenesis [58]. Finally, miR-204-5p was higher in CStC both after osteogenic and EM treatment. It has been documented that miR-204 may act through RUNX2 to inhibit osteogenesis in mesenchymal progenitor cells [59], and expression of miR-204 has been widely recognized has a key factor in vascular remodeling in pulmonary arterial hypertension [60]. Taken together this data, along with the persistent overexpression of miR-146a, may at least partially explained the observed greater propensity of CStC to differentiate into cardiomyocyte-like and endothelial-like cells and their lower adipogenic and osteogenic ability compared to BMStC. Differentiation capacity appears most likely regulated by a complex competitive microRNA network.

Data on miR subsets specifically affecting BMStC differentiation were rather limited. Several signaling pathways, including the bone morphogenetic protein (BMP), Wnt, Hedgehog and insulin-like growth factor pathways, as well as transcription factors such as PPARγ and RUNX2, have been shown to modulate the balance between adipogenesis and osteogenesis [61]. In this respect, it is interesting to note that validated genes modulated by the two miRs (130a-3p and 29a-3p) up-regulated by OM in BMStC, and not in CStC, are involved in the BMP signaling pathway and in the regulation of phosphate metabolic process, osteoblast differentiation and ossification. In addition, Hedgehog is one of the pathways specifically targeted by the BMStC signature. In line with these data, miR-29a has been shown to protect against the adverse actions of glucocorticoid on differentiation capacity of osteogenic cells by regulating β-catenin acetylation [62].

The existence of a tissue molecular signature untouched by *in vitro* treatment has potentially an impact on the development of new reprogramming strategies and is in line with evidences demonstrating that, when a specific combination of miRs efficiently act in reprogramming one type of somatic cells, it might not be so efficient for another cell type [12]. Further, the finding that different, effective differentiating stimuli are not able to erase the expression of signature miRs has potentially great consequences for regenerative medicine, implying that the molecular network sustaining the identity of adult cells is apparently stronger than environmental factors, thus imposing a barrier for the concept of adult cell transdifferentiation. In this light, our evidences strongly suggest that reaching the goal of fully overcoming lineage boundaries should be based necessarily on the knowledge of the main molecular determinants of the cell type at both the starting point and the arrival of the process.

This study has a few limitations. Despite the reliability of the tissue-specific signature, it should be highlighted that CStC and BMStC were exposed to differentiation media and subjected to miR profiling after *in vitro* passaging. For this reason, miR expression levels observed in our conditions may be only partially representative of the *in vivo* condition. Other known cardiac myo-miRs, such as miR-208 [9], were not up-regulated in stromal cells after cardiomyogenic treatment. This could be due to the fact that in vitro drug treatment does not lead to fully differentiated cardiomyocytes. In fact, in our conditions, despite a considerable increase in mRNA expression, only a very low amount of cells (≈0.05–0.1%) stained positive for the protein α-sarcomeric actin [14] and no organized sarcomeric structures were visible after the treatment.

In conclusion, our results demonstrated the existence of a tissue-specific miR signature, which survived to any differentiation stimuli, suggesting that miRs could play a role, along with other several epigenetic factors, in determining cell identity related to tissue origin. Importantly, our results imply that the key factor able to *in vitro* abolish the tissue specific differences still have to be discovered. Moreover, we identified miR subsets modulated by different culture conditions in a cell-specific manner, pointing out their importance during differentiation processes.

## Supporting Information

Table S1 **Average expression levels and analysis of all informative miRs passing filtering criteria.** The table reports the miR expression profiling data, after removal of non informative miRs: *i.e.* (*i*) miR average expression in CStC and BMStC for each culture condition, (*ii*) results of the 2-way ANOVA, and (*iii*) significance levels corrected for multiple comparisons (*q*-values).
(XLSX)
